# Distribution of Arsenic Resistance Genes in Prokaryotes

**DOI:** 10.3389/fmicb.2018.02473

**Published:** 2018-10-23

**Authors:** Ibtissem Ben Fekih, Chengkang Zhang, Yuan Ping Li, Yi Zhao, Hend A. Alwathnani, Quaiser Saquib, Christopher Rensing, Carlos Cervantes

**Affiliations:** ^1^Institute of Environmental Microbiology, College of Resources and Environment, Fujian Agriculture and Forestry University, Fuzhou, China; ^2^Department of Plant and Environmental Sciences, University of Copenhagen, Copenhagen, Denmark; ^3^Department of Botany and Microbiology, King Saud University, Riyadh, Saudi Arabia; ^4^Department of Zoology, College of Sciences, King Saud University, Riyadh, Saudi Arabia; ^5^Key Laboratory of Urban Environment and Health, Institute of Urban Environment, Chinese Academy of Sciences, Xiamen, China; ^6^Instituto de Investigaciones Químico-Biológicas, Universidad Michoacana, Morelia, Mexico

**Keywords:** arsenic, resistance, efflux, mine railings, *ars* operon, remediation

## Abstract

Arsenic is a metalloid that occurs naturally in aquatic and terrestrial environments. The high toxicity of arsenic derivatives converts this element in a serious problem of public health worldwide. There is a global arsenic geocycle in which microbes play a relevant role. Ancient exposure to arsenic derivatives, both inorganic and organic, has represented a selective pressure for microbes to evolve or acquire diverse arsenic resistance genetic systems. In addition, arsenic compounds appear to have been used as a toxin in chemical warfare for a long time selecting for an extended range of arsenic resistance determinants. Arsenic resistance strategies rely mainly on membrane transport pathways that extrude the toxic compounds from the cell cytoplasm. The *ars* operons, first discovered in bacterial R-factors almost 50 years ago, are the most common microbial arsenic resistance systems. Numerous *ars* operons, with a variety of genes and different combinations of them, populate the prokaryotic genomes, including their accessory plasmids, transposons, and genomic islands. Besides these canonical, widespread *ars* gene clusters, which confer resistance to the inorganic forms of arsenic, additional genes have been discovered recently, which broadens the spectrum of arsenic tolerance by detoxifying organic arsenic derivatives often used as toxins. This review summarizes the presence, distribution, organization, and redundance of arsenic resistance genes in prokaryotes.

## Introduction

Arsenic is a metalloid that occurs naturally in aquatic and terrestrial environments. Despite its relatively low abundance in these settings, the high toxicity of arsenic derivatives has converted this element in one of the best studied natural poisons and a severe problem of public health worldwide. This is particularly true by the contamination with arsenic of the groundwater supplies in many countries. Being a human carcinogen, arsenic is considered the most prevalent environmental toxin (Zhu et al., 2014). As it occurs with other biologically relevant chemical elements, a global arsenic geocycle exists, and microorganisms are known to play a crucial role in its functioning (Mukhopadhyay et al., 2002; Zhu et al., 2014).

It is accepted that virtually every organism, from bacteria to humans, has mechanisms for arsenic detoxification, mostly involving transport systems able to extrude arsenite from the cells (Rosen and Liu, 2009). Exposure to arsenic may have started since the beginning of life (Gihring et al., 2003), leading Yang and Rosen (2016) to declare: “Without arsenic detoxification systems life would not exist.”

The more abundant chemical forms of arsenic are the trivalent species, As(III), commonly as the oxyanion arsenite (AsO_2_^−^), and the pentavalent species, As(V), or arsenate (AsO_4_^3−^). Interconversion of these oxyanions occurs in nature with relevant participation of microorganisms; these biotransformations influence the mobilization and availability of arsenic in the environment. Arsenite is more toxic than arsenate because it is able to bind strongly to vicinal sulfhydryl groups in proteins; arsenite also binds weakly to other thiol groups, such as those in glutathione, lipoic acid, and cysteine. Arsenate toxicity is due to its ability to compete with phosphate oxyanions for both transport and energetics functions; the primary toxic effects of arsenate indeed arise from its transformation to arsenite. Organic arsenic compounds, such as arsenic methylated derivatives, also occur in nature and are commonly less toxic than their inorganic counterparts (Mukhopadhyay et al., 2002; Oremland and Stolz, 2003; Stolz et al., 2006). Further details on the abundance, ecology, toxicity and metabolism of arsenic derivatives will not be included in this review as they have been widely reviewed previously (Oremland and Stolz, 2003; Silver and Phung, 2005a; Stolz et al., 2006; Oremland et al., 2009; Páez-Espino et al., 2009; Kruger et al., 2013; Zhu et al., 2014).

## The *ars* Operons

Continuous exposure of organisms to toxic agents in the environment provides a selective pressure to evolve resistance genes. In a similar manner, as it occurred with other toxic heavy metals and metalloids, microorganisms have developed, or acquired, various genetic systems to cope with arsenic toxicity. These systems include the *ars* operons, groups of genes widely distributed in bacterial and archaeal species. *ars* operons frequently occur in most prokaryotic genomes, and it has been stressed that they are more common than genes for tryptophan biosynthesis (Silver and Phung, 2005b). The distribution of arsenic resistance genes is a reflection of the ubiquitous presence of arsenic in nature, but they are present even in microbes isolated from putatively arsenic-free habitats.

In this review, we will focus mostly on the information regarding the presence, distribution, and redundance of prokaryotic genes associated with resistance to arsenic compounds. Details on the biochemical mechanisms for microbial arsenic resistance and their regulation will not be the main subject of this article, as they have been previously reviewed, with different approaches and depth (Mukhopadhyay et al., 2002; Rosen, 2002; Silver and Phung, 2005a; Páez-Espino et al., 2009; Yang et al., 2012; Yang and Rosen, 2016).

The first notion of bacterial genes conferring resistance to arsenic compounds arose 50 years ago from a distant, but related field: the study of antibiotic-resistance genes present in R-factors from clinical bacterial isolates (Novick and Roth, 1968). The *Staphylococcus aureus* pI258 plasmid was found to confer multiple resistances to antibiotics, arsenate, arsenite and other heavy metal derivatives. A few years later, another R-factor, also bearing arsenic resistance genes, the transmissible R773 plasmid, was identified in an *Escherichia coli* strain isolated from a patient with a urinary tract infection (Hedges and Baumberg, 1973). A collaborative research effort thereafter revealed the basic biochemical mechanism of arsenic resistance conferred by the plasmids to their Gram-positive and Gram-negative hosts: the energy-dependent efflux of arsenite from the cell cytoplasm (Mobley and Rosen, 1982; Silver and Keach, 1982).

**FIGURE 1:**
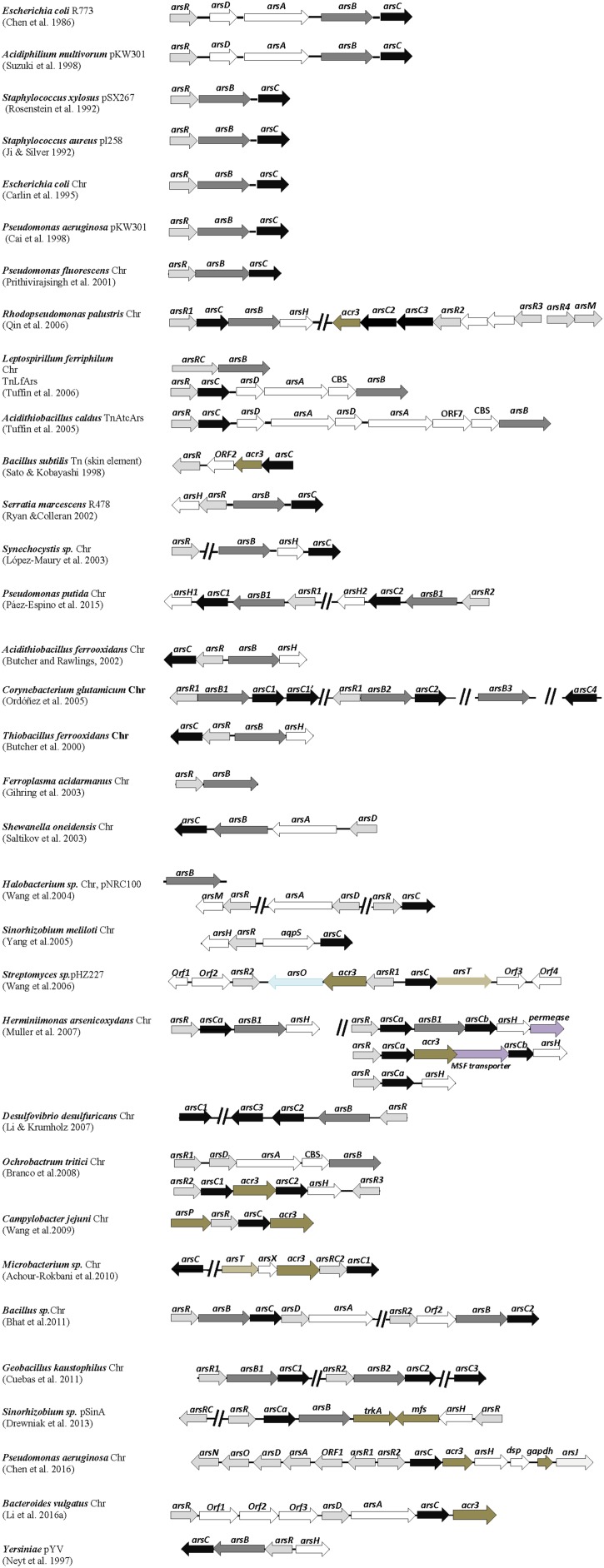
Distribution of *ars* genes in arsenic-resistant prokaryotes. Genetic organization of *ars* operons from various arsenic resistant bacterial strains. Arrows represent open reading frames and orientation of transcription. Chr, chromosomal genes. Gene descriptions and references associated are given in the text.

Shortly before this finding, the energy-dependent efflux of the antibiotic tetracycline had been discovered (McMurry et al., 1980), thus opening the way to the notion that membrane efflux is a common prokaryotic strategy for detoxification of a diversity of compounds, notably heavy metal derivatives (reviewed by Nies, 2003), but also solvent hydrocarbons (reviewed by Ramos et al., 2002). The nucleotide sequence of the determinants from the *E. coli* R773 plasmid identified the *arsRDABC* operon involved in the arsenic resistance phenotype, and staphylococcal plasmids pI258 and pSX267 both contained similar, but simpler *arsRBC* operons encoding proteins with homology to those encoded by R773 (Figure 1). These sequence relationships suggested a similar mechanism of action, as was thereafter confirmed. A brief description of the function of the *ars* operons gene products will follow.

The *arsR* gene encodes ArsR, a member of the SmtB/ArsR family of metalloregulatory proteins (reviewed in Busenlehner et al., 2003). ArsR is a trans-acting transcriptional repressor protein that binds to the promoter region of *ars* operons. The interaction of ArsR with arsenite dissociates the repressor protein from the DNA thus allowing transcription of the operon.

The ArsA protein is an ATPase that interacts with ArsB to form an arsenite efflux pump energized by ATP hydrolysis (reviewed in Yang et al., 2012). Besides its interaction with ArsB, the ArsA ATPase has been proposed to form primary arsenite transporters by association with different membrane proteins (Castillo and Saier, 2010; see below).

ArsB is an integral membrane protein able to extrude arsenite from the cell cytoplasm, thus diminishing arsenite accumulation (reviewed in Yang et al., 2012). ArsB has a dual mode of energy coupling: arsenite efflux may be energized by ATP hydrolysis, catalyzed by ArsA in the complex operons, or by the membrane potential in the *arsRBC* operons, ArsB being driven by the protein motive force.

The ArsC proteins from both the pI258 and R773 plasmids are arsenate reductase enzymes, able to transform arsenate to arsenite prior to extrusion of the latter oxyanion. The ArsC enzymes pertain to two unrelated families: the one from the R773 plasmid uses glutathione and glutaredoxin as electron sources and the enzyme from the pI258 plasmid uses thioredoxin as electron source (reviewed by Zhu et al., 2014). A striking case is the ArsC homolog from the cyanobacterium *Synechocystis* sp. PCC 6803, which shows sequence similarity with the pI258 ArsC enzyme but employs glutathione and glutaredoxin as electron sources (Li et al., 2003).

ArsD functions as an inducer-independent, weak repressor of the *ars* operon, but its primary role is related to its ability to bind arsenite and transfer it to the ArsA ATPase prior to the oxyanion extrusion by the ArsB pump (Lin et al., 2006; reviewed in Yang et al., 2012). ArsD is the first arsenite metallochaperone described.

## Distribution of Prokaryotic *ars* Genes

Besides the mentioned pioneer plasmids R773, pI258 and pSX267, other arsenic resistance plasmids with *arsRBC* or *arsRDABC* gene clusters have been reported (Figure 1). These include plasmids from strains of *E. coli*, the enteric pathogen *Yersinia* spp., *Acidiphilium multivorans* AIU 301, *Serratia marcescens*, the archaea *Halobacterium* sp. NRC-1, and the *Sinorhizobium* sp. M14 strain, isolated from the arsenic-rich soil sediment of a gold mine. Variants of *arsRBC* operons were also identified in transposons from *Bacillus subtilis* strain JH642, the biomining bacterium *Acidithiobacillus caldus* strain f, and the iron-oxidizing bacterium *Leptospirillum ferriphilum* (Figure 1). The presence of arsenic resistance genes on plasmids and transposons constitutes an opportunity for microbes to disseminate these adaptive genetic traits by horizontal gene transfer.

Initial efforts to sequence large fragments of bacterial genomes provided *in silico* evidence of chromosomal *arsRBC* genes in *E. coli*, soon followed by experimental biochemical and molecular data confirming that they function similarly as their plasmid counterparts and a functional *arsRBC* operon was also located in the genome of *Pseudomonas aeruginosa* PAO1 strain (Figure 1). As occurred with other biology fields, the availability of whole genome sequences from diverse prokaryotic species gave rise to a formidable amount of information on the adaptive genes present in those organisms. These included numerous chromosomal *ars* genes from a variety of microbial strains with similarity to those initially identified on plasmids, and these genes are organized in a diversity of configurations in different microorganisms.

Variants of canonical *arsRBC* operons now appear to be quite common in the chromosomes of bacterial and archaeal species of diverse origins, and it is entirely possible that every prokaryotic species has at least one arsenic resistance system. It must be emphasized here that the examples of *ars* genes from the prokaryotic species described in this review (most enlisted in Figure 1) are from reports that involve experimental data on the arsenic resistance phenotype and, in most cases, with gene expression assays, and reports where only gene sequence data are provided were not included. Examples of *ars* operon variants are those identified in *Thiobacillus ferrooxidans*, the marine strain *Pseudomonas fluorescens* MSP3, *Acidithiobacillus ferrooxidans*, *Synechocystis* sp. PCC 6803, the Gamma proteobacterium *Shewanella oneidensis* ANA-3, and the food-borne pathogen *Campylobacter jejuni* (Figure 1). Chromosomal *ars* operons were also identified in the archaeal species *Ferroplasma acidarmanus*, thus suggesting an ancestral origin of arsenic resistance genes.

It has been proposed that simpler *arsRB* operons evolved first on the earth primordial anaerobic environments, where arsenite would be the predominant arsenic oxyanion. Possibily, ancestral gene clusters might encode the ubiquitous ArsR regulator and the ArsB arsenite efflux pump (Rosen, 1999; Mukhopadhyay et al., 2002; Zhu et al., 2014). These minimal *arsRB* operons would allow primitive cells to control the intracellular concentrations of arsenite, thus preventing its toxicity. According to these views, when oxygen appeared in the earth atmosphere, arsenate oxyanions would then be more abundant and ArsC enzymes, able to reduce arsenate to arsenite, evolved, thus giving rise to *arsRBC* operons, whose gene products detoxified arsenite using the preexisting efflux pump. The ArsA ATPase and the ArsD chaperone were probably acquired at a later stage, originating complex *arsRDABC* operons conferring resistance to higher arsenic levels and exerting a tighter regulation (Rosen, 1999). *arsD* and *arsA* genes are nearly always adjacent in *ars* operons from plasmids and chromosomes, suggesting that they act as a unit (Lin et al., 2006).

The *arsBC* gene pair is common in the chromosomes of Gram-negative bacteria and in chromosomes and plasmids of Gram-positive bacteria, but in the latter cases, no *arsA* gene is present. There are examples of *arsC* genes not associated with *arsB* genes in the chromosomes of *P. aeruginosa, Haemophilus influenzae* and *Neisseria gonorrhoeae*. *P. aeruginosa* has a second *arsC* gene besides the one within the *arsRBC* operon (Mukhopadhyay et al., 2002). Interestingly, ArsR-ArsC proteins fusions have been found in several genomes (*L. ferriphilum, Microbacterium, Sinorhizobium*) (Figure 1). It has been suggested that the ArsC proteins seem “predestined” for fusions, given their relatively small size (130–140 amino acid residues) (Wu et al., 2010; see below). If these fusion proteins are functional, they represent an evolutionary advantage to their hosts in terms of arsenite sensing and/or detoxification.

In addition to these simple *ars* operons, Figure 1 shows examples of more complex *ars* gene clusters with a wide variety of gene configurations. Prokaryotes with multiple, redundant *ars* genes appear to be frequent, commonly giving rise to higher levels of resistance to arsenic derivatives (Li and Krumholz, 2007). Redundance of *ars* genes may be the result of gene duplication or horizontal gene transfer. Some microbes possess simple, repeating variants of canonical *ars* operons, such as the *Bacillus* CDB3 strain, isolated from an arsenic-containing cattle dip solution, the thermophilic *Geobacillus kaustophilus* A1 and the soil bacterium *Pseudomonas putida* KT2440. Interestingly, the twin *ars* operons of *P. putida* were found not to function additively in arsenic resistance but rather to express differentially depending on the bacterial growth temperature (Páez-Espino et al., 2015). The archaeal *Halobacterium* sp. possesses *ars* genes in both the main chromosome and in one of its megaplasmids. Similarly, *L. ferriphilum* has *ars* genes in the chromosome and a transposon. Redundant *ars* genes are also present in the Tn*AtcArs* transposon from *A. caldus*. There is a cautionary note that the arsenic resistance levels do not necessarily have a direct correlation with the number of arsenic operons. It is more reasonable to think that prokaryotes with redundant *ars* genes express them in a differential manner depending on the environmental conditions.

Notable examples of bacteria possessing more complex, multiple *ars* genes are the industrially relevant soil bacterium *Corynebacterium glutamicum* ATCC 13032, the anoxygenic phototrophic *Rhodopseudomonas palustris* CGA009, the Beta proteobacterium *Herminiimonas arsenicoxydans*, and the heavy-metal resistant *Ochrobactrum tritici* SCII24 (Figure 1). The redundant *ars* operons from *C. glutamicum* and *R. palustris* express differentially according to the levels of arsenite exposure (Ordóñez et al., 2005; Zhao et al., 2015). Bacterial species with redundant *ars* operons usually inhabit complex, disturbed environments including ecosystems suffering from arsenic contamination.

Some genomes contain tandem repeats of *arsC* genes (*C. glutamicum, R. palustris, D. desulfuricans, Microbacterium* sp.*, Sinorhizobium* sp.) (Figure 1). Interestingly, *Thiomonas* strains isolated from arsenic-containing acid mine drainage possess arsenic genomic islands displaying genes for both arsenic resistance and arsenite oxidation (Freel et al., 2015). “Arsenic gene islands” were first mentioned by Silver and Phung (2005a) referring to groups of genes related to arsenic resistance and arsenic metabolism identified in the soil bacterium *Alcaligenes faecalis*. The pSinA plasmid from *Sinorhizobium* sp. also contains an arsenic genomic island (Figure 1). The presence of genomic islands represents another example of the horizontal transfer of arsenic resistance genes that may contribute to their dissemination.

## Additional Arsenic Resistance Genes

Besides the genes already mentioned for the common *ars* operons, additional genes also involved in arsenic resistance, and commonly linked to *ars* gene clusters, have been identified. This indicates that other arsenic resistance systems exist in prokaryotes, and emphasizes the relevance of arsenic exposure and toxicity in the microbial environments for the development of various arsenic tolerance mechanisms. Included are genes encoding novel inorganic arsenic efflux pumps, such as the Acr3, AqpS, and Major facilitator superfamiliy (MFS) transporters. A brief description of these additional arsenic resistance systems will follow.

The *B. subtilis arsRBC* operon was initially reported to encode a typical ArsB membrane protein (Sato and Kobayashi, 1998). It was later found, however, that the *Bacillus* arsenite efflux pump is indeed a novel transporter with homology to Acr3, a protein encoded by the yeast *Saccharomyces cerevisiae*, which also confers arsenic resistance (Ghosh et al., 1999). The *acr3* gene was subsequently identified in many *ars* gene clusters from diverse bacteria such as *R. palustris*, *H. arsenicoxydans*, *O. tritici*, *C. jejuni*, *Microbacterium* sp., and the obligate anaerobe *Bacteroides vulgatus* ATCC 8482; an *acr3* homolog was also found in the pHZ227 linear plasmid from the arsenic-resistant actinobacterium *Streptomyces* FR-008 strain (Figure 1). Interestingly, in *Mycobacterium tuberculosis*, the Acr3 transporter and an ArsC protein are fused in a single 498 amino-acid polypeptide (Mukhopadhyay et al., 2002).

From a wide genomic analysis, Li et al. (2014) found that Acr3 (also known as ACR3 or ArsY) is the main arsenite efflux pump in the metabolically and ecologically diverse *Burkholderiales* order and this bacterial group includes many arsenic-resistant strains. A different PCR approach, which screened 41 arsenic-resistant soil isolates, revealed the prevalence of *arsB* genes in Firmicutes and Gamma proteobacteria, but a predominance of genes for Acr3 transporters in Actinobacteria and Alpha proteobacteria (Achour et al., 2007). In this study, a phylogenetic analysis displayed two distinct families of Acr3. A similar PCR analysis of 58 arsenic-resistant bacterial isolates from arsenic-contaminated soils showed a predominance of *acr3* genes over *arsB* genes as well as several examples of strains possessing both type of transporters (Cai et al., 2009).

Almost every prokaryotic species has either an *arsB* gene or an *acr3* gene (Yang et al., 2012), in some cases both within a single organism, although no example of the coexistence of the two transporters encoded in the same operon has been reported (Yang et al., 2015). ArsB proteins are present only in prokaryotes, whereas Acr3 proteins are found in bacteria, archaea, fungi and some plants (Castillo and Saier, 2010; Yang et al., 2015). The Acr3 pump may also couple with the ArsA ATPase to form a more efficient primary arsenite efflux system (Rosen, 1999; Castillo and Saier, 2010).

Only limited sequence similarity exists between the ArsB and Acr3 families. Moreover, ArsB has 12 transmembrane domains whereas Acr3 has 10 (Rosen, 1999). The ArsB and Acr3 transporters, in one hand, and the two distinct ArsC arsenate reductase enzyme families on the other, appear to have evolved independently, in an example of convergent evolution, to solve similar problems: to extrude arsenite and to reduce arsenate, respectively (Mukhopadhyay et al., 2002).

The adventitious uptake of arsenate by phosphate transport systems has been long established and these oxyanions share structural properties and arsenate is considered a non-functional analog of phosphate. However, the arsenite transport pathway was unknown until an aquaglyceroporin, the glycerol facilitator GlpF, was discovered in *E. coli* as an “accidental” arsenite uptake transporter (Sanders et al., 1997; Rosen, 2002; Meng et al., 2004). The polyol form of the arsenite oxyanion seems to resemble the structure of its glycerol analog, the natural substrate of GlpF. It is possible that GlpF also functions as an arsenite efflux pump under certain conditions (Bienert et al., 2008; Yang et al., 2012). Aquaglyceroporins have been established as generalized transporters of arsenite and other metalloids (reviewed in Mukhopadhyay et al., 2014). The legume symbiont *Sinorhizobium meliloti* possesses a distinct *ars* operon encoding the aquaglyceroporin AqpS, which may function as an arsenite efflux pump that substitutes for the ArsB transporter (Figure 1). This was the first report linking aquaglyceroporins with arsenic resistance. *S. meliloti* appears to have evolved a distinct mechanism, driven by AqpS, which confers sensitivity to extracellular arsenite but resistance to the arsenite internally generated by reduction of arsenate by the ArsC enzyme (Yang et al., 2005). A striking fusion between an aquaglyceroporin-like transporter and an arsenate reductase is present in the marine actinomycete *Salinospora tropica* CNB-440 (Wu et al., 2010). This novel, bifunctional protein AqpS-ArsC confers the cells the ability to reduce arsenate and to extrude the formed arsenite using a single polypeptide, thus optimizing arsenic detoxification by *S. tropica* cells.

The paired *gapdh* and *arsJ* genes, located in one of the two *ars* operons of *P. aeruginosa* DK2 (Figure 1), confer resistance to arsenate. These genes encode an enzyme, GAPDH (glyceraldehyde 3-phosphate dehydrogenase) and a novel MFS transporter, ArsJ, respectively (Chen et al., 2016). It was proposed that GAPDH catalyzes the formation of an unusual As(V) phosphoglycerate derivative that is then extruded by the ArsJ permease and arsenate would then dissociate from the complex resulting in arsenic detoxification. This ingenious system constitutes an additional, distinct pathway for arsenic resistance, and is the only example reported of a transporter that extrudes arsenate instead of arsenite. The *gapdh* and *arsJ* genes associate with *ars* operons from diverse bacterial species (Chen et al., 2016). Another MFS transporter, encoded by the pSinA plasmid from *Sinorhizobium*, was also involved in arsenic resistance (Drewniak et al., 2013), although a detailed mechanism of action has not yet been reported.

The *arsN* gene was first identified in a metagenomic study from an effluent treatment plant sludge (Chauhan et al., 2009). *arsN* was then found frequently associated with *ars* operons (Chen et al., 2016) and, in some cases, *arsN* sequences are fused with *arsC* or *arsD* genes (Chauhan et al., 2009), thus suggesting a role of the ArsN protein in arsenic resistance. The precise function of ArsN, however, has not been elucidated.

## Resistance to Organoarsenicals

**FIGURE 2:**
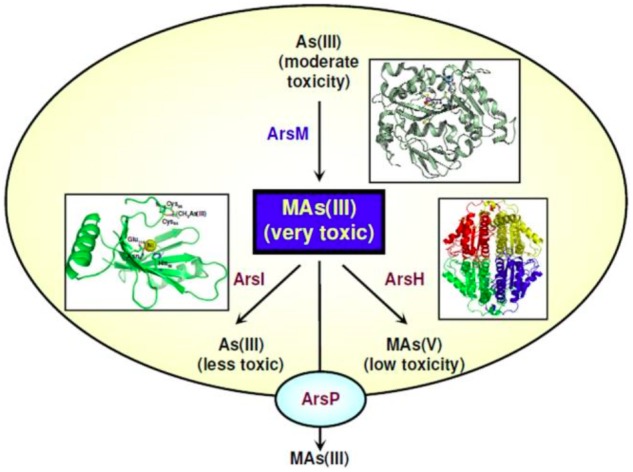
MAs(III): a primordial antibiotic. In communities of soil microbes some bacteria such as *Rhodopseudomonas palustris* carry the *arsM* gene for the As(III) SAM methyltransferase, producing highly toxic MAs(III). This trivalent organoarsenical has antibiotic-like properties. Other soil bacteria carry genes for MAs(III) resistance. Some, such as *Bacillus* MD1, have the *arsI* gene for the ArsI C-As lyase enzyme that confers resistance to MAs(III) by degrading it into As(III) and formaldehyde. Yet other soil bacteria such as *Pseudomonas putida* have a gene encoding ArsH, a flavoprotein that uses NADP+ to oxidize MAs(III) to MAs(V), thus conferring resistance. Finally, other bacteria such as *Campylobacter jejuni*, which inhabits the intestinal track of poultry and other farm animals, carry the *arsP* gene. ArsP is a MAs(III) efflux permease that extrudes trivalent organoarsenicals from cells, conferring resistance. The crystal structures of the relevant enzymes are shown next to their reactions (Li et al., 2016b).

Microbial transformation of organoarsenicals, mainly bacterial arsenic methylation/demethylation, has been known for decades (reviewed in Bentley and Chasteen, 2002), but only recently the molecular details of this process have started to be unveiled. Arsenic methylation is an important part of the global arsenic geocycle, and microorganisms are considered to have important participation in this process (Mukhopadhyay et al., 2002; Oremland and Stolz, 2003). Cycles of arsenic methylation/demethylation are thought to affect the toxicity and availability of arsenic in the environment (Zhu et al., 2014). Arsenic methylation is generally thought of as a detoxification process (Mukhopadhyay et al., 2002; Silver and Phung, 2005a; Qin et al., 2006; Kruger et al., 2013; Zhu et al., 2014; Yang and Rosen, 2016), but not all methylated products are less toxic than the inorganic forms of arsenic (Petrick et al., 2000; Bentley and Chasteen, 2002; Stolz et al., 2006). For example, the aromatic pentavalent arsenical roxarsone is not toxic to bacteria, but the reduced trivalent roxarsone is highly toxic (Chen et al., 2014); also, trimethyl As(III) derivatives are more toxic than inorganic arsenite (Petrick et al., 2000). Therefore, the reduced forms monomethylarsonous acid [MMA(III)], Methylarsenite [MAs(III)] and dimethylarsinous acid [DMA(III)] could also function as highly toxic poisons in the ongoing chemical warfare between organisms (Li et al., 2016b). A genetic system for microbial arsenic methylation related to arsenic resistance was recently characterized (reviewed in Yang and Rosen, 2016). This system includes genes encoding detoxification systems for organoarsenicals, such as the ArsP transporter and the ArsH, ArsM, and ArsI enzymes (Figure 2). The genes and gene products involved in such a system will be briefly described below.

The *arsM* gene, encoding ArsM, an As(III) S-adenosylmethionine methyl transferase enzyme, was first identified in a megaplasmid from the arsenic-resistant archaea *Halobacterium* sp. (Figure 1). This gene was linked to an *ars* operon, and arsenite resistance was lost when *arsM* was deleted (Wang et al., 2004). An *R. palustris* ArsM homolog (Figure 1) was also shown to confer arsenic resistance (Qin et al., 2006). This phenotype was accompanied by the production or trimethyl arsine gas, which suggested that increased volatility of methylated arsenicals overcome the higher toxicity of the intermediates (Qin et al., 2006; Yuan et al., 2008). In a genomic database search, Zhu et al. (2014) found that *arsM* genes are widely distributed in bacteria, suggesting that genes related to arsenic biotransformations evolved early on earth. These authors also proposed that the ArsM enzymes might have a broader role in arsenic detoxification, probably participating in the transformation of other organoarsenical compounds. In contrast, *arsM* genes were not located in the genome of the highly arsenic-resistant *H. arsenicoxydans* (Muller et al., 2007) and were identified only in a few genomes of the Burkholderiales order (out of 188 analyzed) (Li et al., 2014), suggesting that in these bacterial groups arsenic methylation is not a primary pathway for arsenic detoxification. Genes encoding ArsM are often found in operons together with other genes encoding arsenic resistance functions. However in a number of cases, genes encoding ArsM are in operon with only *arsR* encoding the arsenic regulator ArsR.

However, organoarsenicals such as those generated by ArsM in prokaryotes and As3mt in eukaryotes could also act as powerful toxins. This would make resistance systems necessary not only for protection against environmentally occurring organoarsenicals but also against toxins such as MMA(III) or DMA(III) that are much more toxic than inorganic As(III) (Li et al., 2016a). Methylated organoarsenicals are only highly toxic in an anaerobic reducing environment so the toxin can only be effective by close cell to cell contact and keeping a reducing environment under aerobic conditions. The human immune system contains both innate and adaptive immunity. Natural Killer Cells (NKC) are thought to be an evolutionary bridge between these two systems. Interestingly, expression of As3mt was highest in mice NKC compared to other cells in the body, suggesting a possible role of organoarsenicals as toxins in fighting cancer cells, virus-infected cells and pathogenic bacteria (Maimaitiyiming et al., 2018, see link below). http://biogps.org/#goto=genereport&id=57344

The *arsH* gene was first reported in the *ars* operon of the pYV virulence plasmid from *Yersiniae* isolates (Neyt et al., 1997). Interestingly, the presence of an *ars* operon containing *arsH* was correlated to a low virulence phenotype in *Yersinia enterocolitica* but was also found in *Yersinia pestis* strain Java 9. The *arsHRBC* operon was part of transposons Tn2503 (Java 9) and Tn2502 (*Y. enterocolitica*). Pathogenic strains of Yersiniae must fight off attack by both macrophages and NKC cells. We therefore believe resistance against organoarsenicals produced by NKC cells was essential until the gene encoding the plague virulence protein YopM was recruited (Kerschen et al., 2004).

Carbapenem-resistant *Klebsiella pneumoniae* multilocus sequence type 258 has emerged as an important source of hospital death. Plasmid PNJST258N1 (143Kb) contains a 20-gene cluster “copper pathogenicity island” that probably aids in survival in macrophages (Hao et al., 2015) and an *ars* gene cluster also containing *arsH* which might aid in survival in NK cells. Survival in amoeba was enhanced by the presence of an *arsRBC* operon in *E. coli* (Hao et al., 2017). Perhaps this is also true in macrophages. IncHI2 plasmids such as R478 provide increased resistance to arsenate, arsenite, and MMA(III). This *ars* resistance operon (*arsHRBC*) is present in almost all IncHI2 plasmids (Falgenhauer et al., 2017).

Recently, it was found that *arsH* is widely distributed in bacteria, mostly Gamma proteobacteria, but does not occur in Gram-positive bacteria (Páez-Espino et al., 2015). Examples of the presence of *arsH* genes include *ars* gene clusters from the chromosomes of *T. ferrooxidans*, *A. ferrooxidans*, *Synechocystis* sp., *S. meliloti*, *R. palustris*, *H. arsenicoxydans*, *O. tritici*, *P. putida*, and *P. aeruginosa* (Figure 1). Interestingly, the genomes of *H. arsenicoxydans* and *P. putida* have four and two *arsH* genes, respectively. ArsH homologues were also identified in plasmids of *S. marcescens* and of *Sinorhizobium* sp. (Figure 1).

The ArsH protein was demonstrated as an organoarsenical oxidase enzyme conferring resistance to methyl As(III) derivatives in both *P. putida* and *S. meliloti* (Chen et al., 2015a). ArsH oxidizes trivalent organoarsenical compounds to their pentavalent derivatives and thus, along with the ArsM protein (see above), broadens the microbial resistance spectrum of *ars* operons from inorganic to organic arsenicals (Figure 2). The *arsH* gene is also present in the genomes of archaea, fungi, plants, and animals, thus suggesting an ancient origin of the ArsH enzyme (Chen et al., 2015a).

Wang et al. (2009) identified within the *ars* operon of *C. jejuni* a gene encoding a putative membrane transporter, the ArsP permease. ArsP was later suggested as a pump able to extrude the organic arsenical roxarsone (Figure 2) (Shen et al., 2014). The efflux of methyl As(III) and trivalent roxarsone by ArsP was experimentally demonstrated by expressing the *C. jejuni arsP* gene in *E. coli* (Chen et al., 2015b). ArsP did not extrude inorganic arsenite or organic pentavalent arsenicals. ArsP is the first identified efflux system for detoxification of trivalent organoarsenicals (Figure 2), thus widening the efflux pathways of pentavalent organoarsenicals, catalyzed by ArsJ, and of inorganic arsenite, carried out by the ArsB, Acr3 or AqpS transporters.

In a recent genomic sequence search (Yang et al., 2015), ArsP was found widely distributed in bacteria, only surpassed by the Acr3 and ArsB transporters, and ArsP homologs have also been found in archaea and a few eukaryotes, suggesting an ancient origin of the *arsP* gene. In agreement with this divergence, two distinct *arsP* gene clusters occurred in the analyzed bacterial genomes. These groups were not related with the 16S phylogenetic tree and thus probably have different functions (Yang et al., 2015). Acr3 and ArsP transporters coexist in the *C. jejuni ars* operon (Figure 1), where they participate in the extrusion of inorganic and organic arsenicals, respectively (Shen et al., 2014). ArsP has been also proposed to couple with the ArsA ATPase to form a primary arsenite transporter system (Castillo and Saier, 2010), as already suggested for the ArsB and Acr3 transporters, this association might probably increase the ArsP arsenite efflux efficiency. ArsP was shown to give much stronger protection from MMA(III) and DMA(III) than ArsH.

In a genomic sequence survey of *ars* operons from 2,500 bacterial strains, over 700 membrane transporters were identified (Yang et al., 2015). This study revealed five major transporter families, with Acr3 as the most frequent, followed by ArsB, ArsP, and members of the MFS and the Major Intrinsic proteins (MIP) groups. The MIP family includes aquaporins and aquaglyceroporins. These findings further confirmed efflux as the main mechanism of arsenic resistance in prokaryotes.

Organoarsenicals have been largely used as herbicides or pesticides for the maintenance of golf courses as well as for other agricultural, veterinary, and even warfare procedures (Yang and Rosen, 2016). The ArsI enzyme, able to cleave the carbon-arsenic bonds in methylated As(III) derivatives, was first identified in the *Bacillus* sp. MD1 strain isolated from a golf course soil (Figure 2) (Yoshinaga and Rosen, 2014). ArsI is a non-heme iron-dependent deoxygenase with C-As bond lyase activity, and expression of the *Bacillus arsI* gene in *E. coli* conferred resistance to methylated As(III), indicating that demethylation constitutes a detoxification process. *arsI* genes are widely distributed in aerobic bacteria, where they appear to occur always within *ars* operons (Yoshinaga and Rosen, 2014). It was proposed that ArsI activity plays an important role in the global arsenic geocycle. Another ArsI homologue, from the freshwater cyanobacterium *Nostoc* sp. PCC 7120, was recently characterized (Yan et al., 2015). The enzyme also conferred resistance to methylated As(III) and was able to demethylate both As(III) and As(V) derivatives.

Genes encoding the predicted proteins ArsO and ArsT were also frequently found as part of an operon involved with handling arsenicals (Wang et al., 2006). However, their exact functions have not been deciphered yet.

Arsenic in the environment is not only present as arsenite (AsIII) and arsenate (AsV) but also as thioarsenates under sulfur-reducing conditions (Stauder et al., 2005). In the presence of sulfate, sulfate-reducing bacteria outcompete methanogens under anaerobic conditions producing hydrogen sulfide, H_2_S. Geological processes can also lead to the presence of various forms of sulfur and subsequently formation of thioarsenates (H_x_As^V^S^−II^_n_O_4−n_^3−x^; n = 1-4; x = 1-3) (Besold et al., 2018). A sulfate reducing Gram-positive, obligately anaerobic soil bacterium, *Desulfotomaculum* TC-1, was shown to be able to couple anaerobic arsenite oxidation to production of arsenate, which can then be converted into thioarsenate, thereby linking these two processes (Planer-Friedrich et al., 2015; Wu et al., 2017). Interestingly, volcanic thermal vents often contain high concentrations of sulfur and arsenic, possibly mimicking conditions of early life (Hug et al., 2014). This implies that there was continuous presence of thioarsenates throughout Earth’s history. The presence of the various thioarsenates also suggests that mechanisms for thioarsenate resistance should exist (Planer-Friedrich et al., 2008). Microbial resistance could employ reduction and subsequent efflux, although this has yet to be verified.

Of interest is also the presence of methylated thioarsenicals, which are extremely toxic. For example, DMMTAs(V) (dimethylmonothioarsinic Acid) is more toxic than DMA(V) (dimethylarsinic acid) (Naranmandura et al., 2007). AS3MT is required for production of urinary methylated thioarsenates and oxythioarsenates in mice, indicating that As(III) methylation is a key step in formation of methylated thioarsenates (Kanwal and Hua, 2013). Biomethylation of arsenite is usually attributed to the catalytic activity of ArsM/AS3MT. However, methylation of arsenite also occurs independent of ArsM. Methanogens are able to methylate metalloids using methylcobalmin [CH_3_Cob(III)], 2-mercaptoethanesulfonic acid (CoM) and the methyltransferase MtaA as a side reaction of methanogenesis (Thomas et al., 2011). This is a completely different mechanism from ArsM-catalyzed methylation of As(III). ArsM is regulated by the As(III)-responsive repressor ArsR and is thus thus dependent on the presence of arsenite. The alternate methylation reaction may be regulated differently and may not require As(III), although this has not been demonstrated.

## Concluding Remarks

**FIGURE 3:**
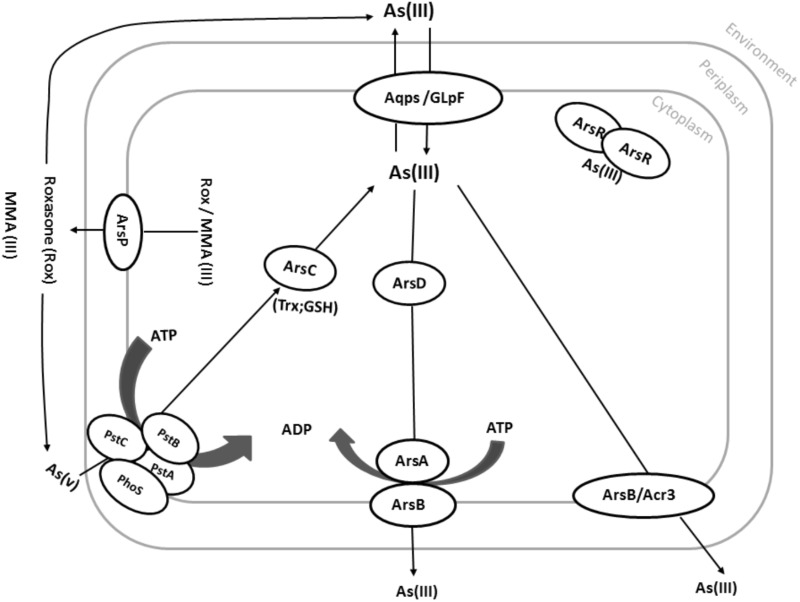
Common pathways in arsenic resistance on prokaryotes. Under aerobic conditions, As(V) enters the cell via phosphate uptake systems (here PstA, PstB, PstC, and PhoS). As(V) is then reduced by the arsenate reductase ArsC to As(III). Although As(III) is more toxic than As(V), As(III) can easily be distinguished from phosphate, which is very similar to As(V). As(III) can also directly be taken up by various aquaglyceroporins such as GlpF from *E. coli*. As(III) can then be translocated across the cytoplasmic membrane via Acr3 or ArsB using the proton motive force (PMF). Alternatively, As(III) can be bound by the As(III)-binding chaperone ArsD and delivered to the ATP-dependent ArsAB efflux pump. Organic arsenic compounds such as MMA(III) and Roxarsone can also be pumped out by the ArsP transporter.

Exposure of microorganisms to arsenic compounds in the environment, possibly since the beginning of life, has provided a strong elective pressure for them to evolve various genetic systems to cope with arsenic toxicity. These systems include mainly the ubiquitous *ars* operons, devoted to inorganic arsenic detoxification, but additional genes enrich the prokaryotic genomes and widen the arsenic resistance spectrum by including the toxic, organic forms of arsenic (Figure 3). Most biotransformations of arsenic involve microbes as active participants in the global arsenic geochemical cycle. The ubiquitous nature of *ars* genes in the microbial world clearly illustrates their ancient origin and suggests that they were present in the most primitive prokaryotic genomes. The location of arsenic resistance genes on plasmids, transposons and genomic islands emphasizes the involvement of horizontal gene transfer processes as efficient mechanisms of gene dissemination.

## Author Contributions

IBF and CZ wrote part of the review. YL, YZ, HA, and QS reviewed and edited the review. CR wrote the review. CC wrote and conceptualized the review.

## Conflict of Interest Statement

The authors declare that the research was conducted in the absence of any commercial or financial relationships that could be construed as a potential conflict of interest.
